# Parental styles are associated with eating disorder symptoms, anxiety, interpersonal difficulties, and nucleus accumbens response

**DOI:** 10.1007/s40519-024-01684-2

**Published:** 2024-08-31

**Authors:** Neha Sahota, Megan E. Shott, Guido K. W. Frank

**Affiliations:** Department of Psychiatry, University of California, 4510 Executive Drive #315, San Diego, CA 92121 USA

**Keywords:** Eating disorder, Parental bonding, Parenting style, Dopamine, Nucleus accumbens, Development

## Abstract

**Objectives:**

Eating disorders (EDs) typically emerge during adolescence. Parental bonding has a lasting impact on a child’s mental health during those developmentally critical years. There remains uncertainty over whether parental bonding is a risk factor for developing or maintaining specifically EDs or, rather, general psychopathology and the associated underlying brain function.

**Methods:**

Forty-one young adult healthy control individuals (HC, 26.6 ± 3.5 years) and 46 individuals with EDs (25 with anorexia nervosa, AN, 22.8 ± 6.4 years, and 21 with bulimia nervosa, BN, 23.5 ± 4.2 years) completed the parental bonding instrument (PBI), assessments for anxiety, depression, and ED behaviors, and underwent a conditioning paradigm during brain imaging.

**Results:**

In both groups, perceived parental care and overprotection were correlated with state and trait anxiety and interpersonal alienation, and in HC only, with body dissatisfaction and drive for thinness. Individuals with an ED reported lower self-perceived parental care, but higher overprotection compared to the HC group. Nucleus accumbens (NAc) response was related to bonding measures in both groups and right NAc response mediated the relationship between maternal care and trait anxiety in HC.

**Conclusions:**

Perceived parental bonding is associated with general psychopathology, including elevated anxiety and interpersonal difficulties across HC and ED groups. Lower perceived parental care and higher overprotection could predispose healthy individuals to develop problems with body shape or weight; however, other, maybe biological factors may determine whether a person will develop an ED. The link between perceived parental bonding, NAc valence processing and anxiety implicates dopaminergic circuits that should be studied further.

*Level of Evidence*: Level III: Case–control analytic study

**Supplementary Information:**

The online version contains supplementary material available at 10.1007/s40519-024-01684-2.

## Introduction

Eating disorders (EDs) such as anorexia nervosa (AN) and bulimia nervosa (BN) are severe psychiatric illnesses. The etiology of EDs is considered multifactorial with complex bio-psycho-social underpinnings [1, 2].

Whether parenting styles can be directly linked to ED psychopathology or are primarily risk factors for general psychopathology is unclear [3]. One facet of parenting styles, parental bonding, the perceived maternal and paternal parenting dynamic based on reported levels of 'care' and 'overprotection or control' from the child's perspective, has been assessed using the parental bonding instrument (PBI) in various studies [4, 5]. One study, for instance, reported that parental bonding was associated with a high drive for thinness in healthy controls, and the “affectionless control” parenting style was more common in individuals with EDs [6], suggesting that maternal or paternal parenting style might play a role in ED development. However, a cross-cultural study did not indicate that parental bonding is predictive of ED pathology, while others suggested that it may matter for certain subtypes [7, 8]. Some literature reported that higher-weight individuals perceive parents (specifically fathers) as more controlling and less caring [9]. A retrospective study on adolescents with and without an ED showed that parental care was predictive of general psychopathology but did not indicate that PBI parenting styles were associated with an AN diagnosis [10]. Interestingly, one study reported on lower perceived parental care in individuals chronically ill with AN, while a group recovered from AN did not show this pattern [11]. One possible explanation is that an individual’s perception of parenting style may shift between the state of illness and recovery. In summary, the literature on the interaction of parenting styles and ED behaviors or development is small and inconsistent, warranting further study.

Neurobiologically, the effects of parental bonding have been associated with dopamine neurotransmission. For instance, in humans, lower parental care was associated with higher dopamine release when stressed, and rodents raised by two parents had more nucleus accumbens (NAc) dopamine turnover than mono-parentally raised offspring [12–14]. Parental care thus may play an important role in shaping reward valence or salient stimulus processing. Extremes of low or high food intake are also associated with altered dopamine-related brain responses, and parental style during childhood could further shape this response and affect eating behavior [15, 16]. In summary, parental care has been implicated in dopaminergic neurotransmission; however, the connection with ED behavior expression or development is less clear.

In this exploratory study, we applied the PBI to young women with AN or BN and a control group who also underwent a dopamine-associated taste learning paradigm during brain imaging. We expected that ED-related behaviors would be associated with elevated perceived parental control and lower care across groups. We further tested the hypothesis that lower perceived parental care and higher overprotection or control would be associated with stronger dopamine-related brain response.

## Methods

### Participants and procedures

We recruited women with AN (*n* = 25), BN (*n* = 21), and healthy control women (HC) (*n* = 41). The HC group was recruited from the community, and the AN and BN groups were recruited from a specialized ED treatment program and the community. All participants were assessed with the Structured Clinical Interview for DSM-5 Disorders by a doctoral-level interviewer. This was a secondary analysis of a larger sample that only in part had completed the PBI [17].

Written informed consent was obtained prior to participation, and the local Institutional Review Board approved all study procedures.

### Assessments

Parental Bonding Instrument (PBI): The PBI is an assessment designed to measure maternal and paternal parenting styles from the child’s perspective after the age of 16 years [5]. Outcome measures are reported on a scale of “care” and “overprotection," resulting in four measured variables (maternal care, paternal care, maternal overprotection, and paternal overprotection). This instrument provides a standard and reliable way of assessing parenting style on a scale of protection and care levels [12]. From these scale scores, parents can be categorized into a specific parenting style, each categorized by a combination of “high” or “low” care and overprotection. A singular cutoff score for each parent determines this designation. A care score of 27 and 24 for mothers and fathers, respectively, delineates whether a parent is considered high on care (exceeding the cutoff) or low on care (beneath the cutoff). The cutoff for protection for mothers and fathers is a score of 13.5 and 12.5, respectively, to categorize a parent as high or low on protection [18]. There are four parenting styles derived from the PBI, three of which are “Neglectful” (low care and low protection), “Optimal” (high care and low protection), and “Affectionate Constraint” (high care and high protection). Previous literature has reported that the most common parenting style in individuals with eating disorders is “Affectionless Control'', characterized by a high protection score and low care score [18].

To assess the reliability of the index, we calculated Cronbach's Alpha for maternal overprotection, maternal care, paternal overprotection, and paternal care. We found *α* = 0.944, 0.886, 0.953, and 0.886, respectively.

Other assessments applied were the Beck Depression Inventory-II (BDI), State–Trait Anxiety Inventory (STAI), and the Eating Disorder Inventory (EDI-3). The analysis included those measures to test the relationships between PBI measures and general versus ED-specific psychopathology. The Supplemental Information provides a complete description of those assessments.

### Reward circuit brain imaging

Brain imaging results from a larger sample were reported previously indicating that ED behaviors alter brain reward or salient stimulus processing [17]. Here, the goal was to explore and test the association of brain response with PBI measures using the subset that had completed both brain imaging and PBI. Data were analyzed for the dopamine-related prediction error response to track stimulus salience response [19]. In addition, unexpected stimulus receipt and omission responses (positive or negative surprise) were analyzed separately [19].

fMRI Image Acquisition. Between 0700 and 0900 h, participants ate a standard breakfast. Then, brain imaging was performed between 0800 and 0900 h on a 3 T GE Signa scanner (three-plane scout scan 16 s) for sagittally acquired, spoiled gradient sequence T1-weighted, 172 slices, thickness = 1 mm, TI = 450 ms, TR = 8 ms, TE = 4 ms, flip angle = 12°, FOV = 22 cm, scan matrix = 64 × 64, and T2*-weighted echo-planar scans for blood-oxygen-level-dependent (BOLD) functional activity (3.4 × 3.4 × 2.6 mm voxels, TR = 2100 ms, TE = 30 ms, flip angle = 70 °, 28 axial slices, thickness = 2.6 mm, gap = 1.4 mm).

Taste Reward Task [20]. Participants learned to associate three unconditioned taste stimuli (US: 1 M sucrose solution, no solution, or artificial saliva) with paired conditioned visual stimuli (CS). Each CS was probabilistically associated with its US such that 20% of sucrose and no solution CS trials were unexpectedly followed by no solution and sucrose US, respectively. Taste stimuli were applied using a customized programmable syringe pump (J-Kem Scientific, St Louis, MO, USA) and E-Prime Software (PST, Pittsburgh, PA, USA) [21].

fMRI Data Preprocessing. Image preprocessing and analysis were performed using SPM12 (http://www.fil.ion.ucl.ac.uk/spm/software/spm12/). Images were realigned to the first volume, normalized to the Montreal Neurological Institute template, and smoothed at 6 mm full width at half maximum Gaussian kernel. Data were preprocessed with slice time correction and modeled with a hemodynamic response convolved function using the general linear model, including temporal and dispersion derivatives. A 128-s high-pass filter was applied for low-frequency BOLD signal fluctuations and motion parameters as first-level analysis regressors.

Data were analyzed for prediction error and unexpected sucrose receipt and omission, described in detail in the Supplemental Material.

Region of Interest (ROI) Data Extraction. We extracted parameter estimates (prediction error analysis) and beta values (group-by-condition analyses) from the NAc to test comparability with previous studies [12–14, 22].

### Statistical analysis

Statistical analyses were performed using the IBM Statistical Package for Social Sciences (SPSS Version 28.1). Data were non-normally distributed, and a Kruskal–Wallis test for multiple independent samples was used for a non-parametric assessment of the distribution of PBI scores across the AN, HC, and BN groups and Bonferroni corrected post-hoc group comparison. Spearman correlation assessed relationships between study variables and analyses were false discovery rate (FDR) corrected. For the correlation analyses, the data set was separated into the healthy control group (*n* = 41) and the ED group (*n* = 46), consisting of AN and BN participants. A Fisher-Z transformation was conducted on rank-transformed data to compare the correlation strength between the two groups. SPSS PROCESS was used to conduct exploratory mediation analyses, and results were bootstrapped (5000 samples).

## Results

### Demographic group characteristics

Compared to the HC group (Supplemental Table 1), the AN and BN groups were younger, but the overall age range was narrow. BMI was lower in the AN group compared to the HC and BN groups. Both the AN and BN groups had higher state and trait anxiety values, BDI, and EDI-3 values (bulimia, body dissatisfaction, drive for thinness, low self-esteem, personal alienation, interpersonal insecurity, interpersonal alienation, interoceptive deficits, emotional dysregulation, perfectionism, asceticism, maturity fears), and binge/purge frequency.

### PBI scores group comparisons

Perceived maternal care was lower in AN (*p* < 0.05) and BN (*p* < 0.001) compared to the HC group; perceived maternal overprotection was higher in BN compared to the HC group (p < 0.05); perceived paternal care was lower in AN (*p* < 0.05) and BN (*p* < 0.05) compared to the HC group; perceived paternal overprotection was higher in AN (*p* < 0.05) and BN (*p* < 0.05) compared to HC (Supplemental Table 1, Supplemental Fig. 1).

### Correlation analyses—PBI scores and behavioral data

**Fig. 1:**
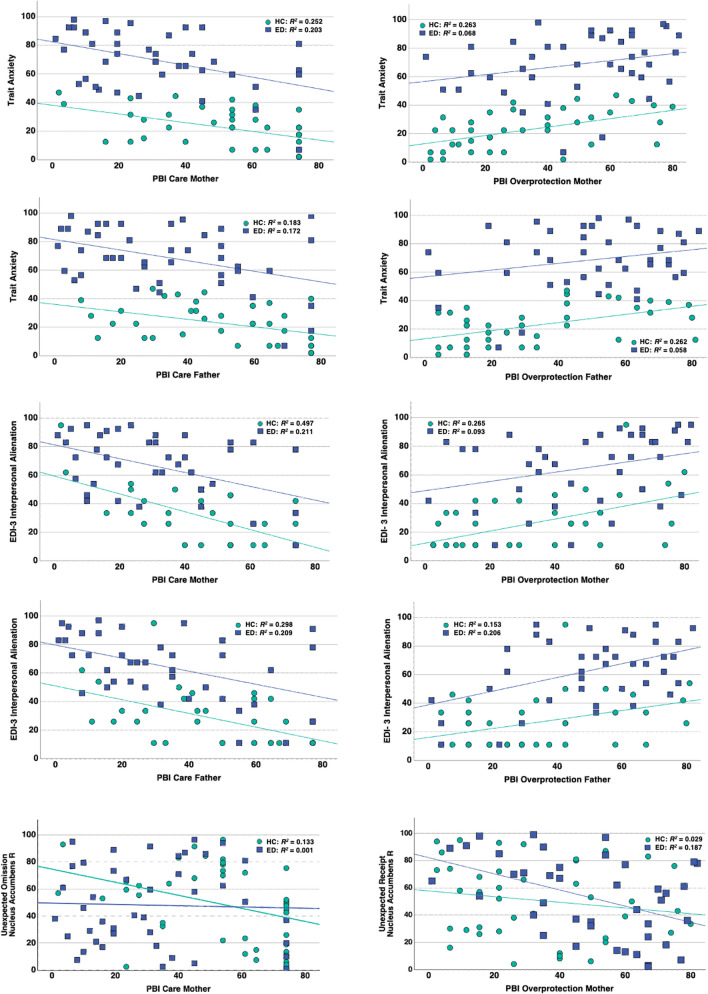
Correlation graphs for the Parental Bonding Instrument (PBI) variables, behavior, and nucleus accumbens (NAc) activation

In the HC group (Table 1, Fig. 1), after FDR correction, there was a statistically significant negative correlation between maternal care and body dissatisfaction, drive for thinness, bulimia, interpersonal insecurity, interpersonal alienation, state anxiety, trait anxiety, and depression scores; paternal care showed a significant negative correlation with interpersonal alienation and trait anxiety in healthy controls. Maternal overprotection in the HC group had a statistically significant positive correlation with body dissatisfaction, drive for thinness, interpersonal insecurity, interpersonal alienation, asceticism, state anxiety, and trait anxiety. Paternal overprotection showed a statistically significant positive correlation with body dissatisfaction, drive for thinness, state anxiety, trait anxiety, and higher depression scores.

**Table 1:** Spearman correlation between the Parental Bonding Instrument (PBI) metrics and assessments measuring eating and other psychiatric psychopathology

	Maternal care				Maternal OP				Paternal care				Paternal OP			
	HC		ED		HC		ED		HC		ED		HC		ED	
	rho	*p*	rho	p	rho	*p*	rho	p	rho	*p*	rho	p	rho	*p*	rho	p
Body dissatisfaction	-0.48	0.002^a^	-0.14	0.353	0.56	< 0.001^a^	0.28	0.059	-0.33	0.035	-0.16	0.288	0.61	< 0.001^a^	0.11	0.467
Drive for thinness	-0.38	0.015^a^	0.16	0.288	0.46	0.002^a^	-0.06	0.692	-0.25	0.115	0.00	1.000	0.45	0.004^a^	0.06	0.692
Bulimia	-0.41	0.008^a^	-0.22	0.142	0.33	0.033	0.34	0.021	-0.34	0.030	-0.13	0.389	0.34	0.029	0.28	0.059
Low self-esteem	-0.31	0.049	-0.39	0.008^a^	0.21	0.188	0.16	0.288	-0.19	0.234	-0.24	0.108	0.22	0.167	0.25	0.094
Personal alienation	-0.30	0.057	-0.28	0.059	0.30	0.057	0.24	0.108	-0.17	0.288	-0.31	0.003^a^	0.22	0.167	0.34	0.020
Interpersonal insecurity	-0.43	0.005^a^	0.00	1.000	0.49	0.001^a^	0.13	0.389	-0.33	0.035	-0.07	0.644	0.35	0.025	0.13	0.389
Interpersonal alienation	-0.63	< 0.001^a^	-0.38	0.010^a^	0.48	0.001^a^	0.36	0.014	-0.60	< 0.001^a^	-0.44	0.002^a^	0.36	0.022	0.34	0.023
Interoceptive deficits	-0.23	0.148	-0.19	0.206	0.25	0.115	0.04	0.792	-0.07	0.664	-0.09	0.552	0.25	0.115	0.15	0.320
Emotional disregulation	-0.25	0.115	-0.31	0.037	0.13	0.418	0.34	0.022	-0.16	0.318	-0.31	0.035	0.17	0.288	0.39	0.008
Perfectionism	-0.04	0.804	-0.07	0.644	0.09	0.576	0.00	1.000	-0.12	0.455	-0.20	0.183	0.03	0.823	0.25	0.094
Asceticism	-0.24	0.131	0.07	0.644	0.38	0.016	0.00	1.000	-0.15	0.349	-0.01	0.947	0.20	0.210	−0.13	0.389
Maturity fears	-0.31	0.049	-0.10	0.508	0.25	0.115	0.22	0.142	-0.17	0.288	-0.39	0.008^a^	0.18	0.260	0.32	0.030
State anxiety	-0.37	0.018^a^	-0.39	0.007^a^	0.53	< 0.001	0.25	0.094	-0.22	0.167	-0.33	0.026	0.39	0.012^a^	0.15	0.320
Trait anxiety	-0.52	< 0.001^a^	-0.41	0.005^a^	0.53	< 0.001	0.32	0.031	-0.47	0.002^a^	-0.34	0.023	0.51	0.001^a^	0.13	0.389
Depression	-0.38	0.014^a^	-0.36	0.014^a^	0.32	0.040	0.35	0.018	-0.24	0.131	-0.30	0.043	0.39	0.012^a^	0.28	0.059

^a^indicates significance after false discovery rate (FDR) correction

The ED group showed statistically significant negative correlations between maternal care and low self-esteem, interpersonal alienation, state anxiety, trait anxiety, and depression. In addition, there was a significant negative correlation between paternal care and personal alienation, interpersonal alienation, and maturity fears (Table 1, Fig. 1). There were no statistically significant correlations between parental care or overprotection and ED-specific symptoms in this group.

A Fisher-Z transformation did not indicate significant group differences with respect to correlation slopes for those associations.

### Correlation analyses—PBI, ED behaviors, and brain imaging data

NAc prediction error response was not related to PBI values in either group. In HC, NAc response to unexpected, sweet stimulus omission was inversely associated with maternal care on the right (rho = −0.42, *p* = 0.007) and on the left (rho = −0.04, *p* = 0.022), which remained significant after FDR-correction on the right side (*q* = 0.042). Right-sided NAc response to unexpected, sweet stimulus omission was also related to trait anxiety (rho = 0.34, *p* = 0.030) and body dissatisfaction (rho = 0.401, *p* = 0.009). We therefore explored whether the direct effect of maternal care on body dissatisfaction and trait anxiety was mediated by NAc response. The maternal care–body dissatisfaction relationship was not mediated by NAc response; however, the maternal care–trait anxiety relationship was partially mediated. The mediation model (Supplemental Fig. 2) indicated significant correlations between maternal care and right NAc response (a, coefficient = -0.49, *p* = 0.030), NAc and trait anxiety (b, coefficient = 0.14, *p* = 0.032), and a direct effect of maternal care on trait anxiety (c’, coefficient = −0.24, *p* = 0.010) that was partially mediated by NAc response (indirect effect, coefficient = −0.07, LLCI95% = -0.18, ULCI95% = −0.003).

In the ED group, NAc activation to unexpected, sweet stimulus receipt was inversely related to maternal overprotection on the right r = −0.43, p = 0.005) and on the left (r = −0.31, p = 0.049), which remained significant on the right side (*q* = 0.029). NAc response did not mediate the maternal overprotection–trait anxiety relationship.

## Discussion

This study indicates that only in the HC group is maternal care negatively and overprotection positively related to EDI body dissatisfaction, drive for thinness, and bulimia. In both study groups, perceived maternal care was significantly negatively and overprotection positively associated with trait anxiety and interpersonal alienation. EDs were associated with lower perceived parental care and higher perceived parental overcontrol. In the HC group, NAc response to unexpected stimulus omission partially mediated the association between maternal care and trait anxiety.

While the ED groups presented scores lower on parental care and higher on overprotection or control, those scores were not correlated with ED behaviors. In contrast, in the HC group, maternal care and maternal and paternal overprotection were significantly correlated with their scores for body dissatisfaction and drive for thinness. Because the EDI-3 scales are considered “risk scales,” it is possible that maternal care and overprotection constitute a risk for developing ED symptoms in individuals who have not developed an ED. However, in those who develop an ED, biological factors may become the driving forces. Consistent with previous research, it is possible that low perceived parental care and elevated perceived parental overcontrol are state or acute illness-dependent and may normalize with recovery [11].

Maternal care was negatively correlated with state and trait anxiety, depression, and interpersonal alienation scores in both study groups. Interpersonal alienation measures feelings of estrangement and a lack of trust and understanding in relationships. Paternal care and parental overprotection had some but less consistent associations with anxiety or interpersonal difficulties across groups. Those results highlight the importance of parental bonding and especially maternal care on anxious traits, mood, and relational problems.

The dopamine-associated prediction error response was not associated with parental bonding measures in either group, which was unexpected. However, higher maternal care in HC was associated with a higher degree of the expected negative right NAc response to unexpected, sweet stimulus *omission*. This result could indicate more sensitivity to loss and disappointment in HC brought up with high care and affection. Positive NAc brain response is expected to unexpected stimulus *receipt*, and lower right-sided response in the ED group in relation to maternal overprotection may be interpreted as such that this group could have lower ability to enjoy or respond to receiving potential rewards in relation to perceived maternal overprotection or control. It is possible that maternal control or overprotection can shape specific brain, including NAc responses in HC and ED groups, which may have long-lasting effects on anticipatory or consummatory pleasure across different domains. The exploratory mediation analysis suggests that in the HC group, the relationship between maternal care and trait anxiety is partially mediated by NAc response, supporting previous research that linked brain dopamine function with trait anxiety [23].

### Strengths and limitations

The study sample was modest. However, the existing effect sizes for maternal care, maternal overprotection, paternal care, and paternal overprotection were medium to large, with eta-squared values of 0.19, 0.08, 0.09, and 0.11, respectively. The PBI has limitations as a self-reported, retrospective assessment but has shown to be a relatively reliable source of information for evaluating this parental behavior [24]. Whether the link between parenting style, dopaminergic circuit response and ED behaviors is established for instance due to conditioning requires further study. Another future research direction should be whether altered dopamine brain response during the ill state of an ED changes perceived parental care.

## Conclusion

The study suggests that lower perceived parental bonding, especially maternal care, is associated with state and trait anxiety, low mood, and interpersonal difficulties across study samples. Lower care and higher overprotection may predispose to body dissatisfaction and drive for thinness in healthy individuals, but in individuals who developed an ED, other maybe biological factors may determine whether a person will develop an ED or perpetuate the ED psychopathology. The diverging relationships between NAc response to unexpected stimulus omission or receipt in the HC and ED groups raises questions about different neurobiology-behavior associations during development across those individuals that should be investigated further.

## What is known on this subject?

- Several studies found a significant association between eating disorder diagnosis and a self-perceived “affectionless control” parenting style.
- Eating disorders are associated with altered dopamine processing.
- Parental bonding is linked to variations in dopamine release from the NAc.

## What does the study add?

- In the eating disorder and healthy control participants, parental care and overprotection correlated with state and trait anxiety, depression and interpersonal difficulties, indicating effects on general psychopathology.
- The association between parenting style and eating disorder psychopathology in healthy controls could indicate a risk for developing an eating disorder. There was no indication that parental bonding is associated with eating disorder specific behaviors in individuals who have developed anorexia or bulimia nervosa, and biological factors may be more relevant in that group.

### Supplementary Information

Below is the link to the electronic supplementary material.

Supplementary Material 1.

## Data Availability

Data are available upon reasonable request.
