# Supplementary material for: Parental styles are associated with eating disorder symptoms, anxiety, interpersonal difficulties, and nucleus accumbens response
Source: Eat Weight Disord. 2024 Aug 31;29(1):55. doi: 10.1007/s40519-024-01684-2 (PMC11365925; doi:10.1007/s40519-024-01684-2)
Supplement: Supplementary file 1 — Supplementary Material 1. [file 40519_2024_1684_MOESM1_ESM.docx]

**Parental styles are associated with eating disorder symptoms, anxiety, interpersonal difficulties, and nucleus accumbens response.**

**Supplemental Information**

Assessments

Parental Bonding Instrument (PBI): The PBI is an assessment designed to measure maternal and paternal parenting styles from the child's perspective after the age of 16 years old [4]. Outcome measures are reported on a scale of “care” and “overprotection," resulting in 4 measured variables (maternal care, paternal care, maternal overprotection, and paternal overprotection). This instrument provides a standard and reliable way of assessing parenting style on a scale of protection and care levels (12). From these scale scores, parents can be categorized into a specific parenting style, each categorized by a combination of "high" or "low" care and overprotection. A singular cutoff score for each parent determines this designation. For mothers and fathers, a care score of 27 and 24 delineates whether a parent is considered high on care (exceeding the cutoff) or low on care (beneath the cutoff). The cutoff for protection for mothers and fathers is a score of 13.5 and 12.5, respectively, to categorize a parent as high or low on protection [11]. There are four parenting styles derived from the PBI, three of which are “Neglectful” (low care and low protection), “Optimal” (high care and low protection), and “Affectionate Constraint” (high care and high protection). Previous literature has reported that the most common parenting style in individuals with eating disorders is “Affectionless Control'', characterized by a high protection score and low care score [11].

To assess the reliability of the index, we calculated Cronbach's Alpha for maternal overprotection, maternal care, paternal overprotection, and paternal care. We found α= .944, .886, .953, and .886, respectively.

Beck Depression Inventory-II (BDI) is a 21-question inventory to assess the level and severity of depression symptoms [12].

State-Trait Anxiety Inventory (STAI) measures state and trait anxiety in participants, separate from depressive symptoms [13].

Eating Disorder Inventory (EDI-3) is a self-reported inventory assessing ED behaviors [14], including DT: Drive for Thinness and preoccupation with weight. B: Bulimia, a tendency to binge eat and purge. BD: Body dissatisfaction, view of one’s own shape/size. LSE: Low self-esteem. PA: Personal alienation, lack of understanding, or emotional comfort with oneself. II: Interpersonal Insecurity, insecurity in social settings. IA: Interpersonal Alienation, perceived alienation in social situations. ID: Interoceptive Deficits, self-awareness of one’s own emotions. ED: Emotional dysregulation, mood stability. P: Perfectionism is a person’s own standards and adherence to them. A: Asceticism, self-discipline, and restraint from pleasure. MF: Maturity Fears, concern about developing out of childhood.

Prediction Error Analysis. Each participant’s prediction error signal was modeled based on trial sequence and regressed with brain activation across all trials [18, 20, 21]. The predicted value ($\hat{V}$) at any time (*t)* within a trial is calculated as a linear product of weights (*w_i_)* and the presence of a conditioned visual stimulus (CS) at time *t,* coded in a stimulus representation vector *x_i_(t)* where each stimulus *x_i_* is represented separately at each moment in time:

$$V\left( t \right)= \sum_{i} W_{i}x_{i}\left( t \right)$$

Predicted stimulus value at time *t* is updated by comparing the predicted value at time *t*+1 to that actually observed at time *t,* leading to the prediction error *δ(t)*:

$$\delta\left( t \right)= r\left( t \right)+ \gamma\hat{V}\left( t+1 \right)-\hat{V}(t)$$

where *r(t)* is the reward at time *t.* The parameter *𝛾* is a discount factor, which determines the extent to which rewards arriving sooner are more important than rewards that arrive later during the task, with $\gamma$=0.99. The weights w_i_ relate to how likely a particular unconditioned reward stimulus (US) follows the associated CS and are updated on each trial according to the correlation between prediction error and the stimulus representation:

$$\Delta w_{i}= \alpha\sum_{t} x_{i}\left( t \right)\delta\left( t \right)$$

where α is a learning rate. A slow α=0.2 was applied (see supplemental material). Initial reward values were 1 for Sucrose Receipt and 0 for No Sucrose. Trial-to-trial prediction error was regressed with brain activation across all trials within each subject. The prediction error calculated for each trial was modeled as an absolute (reflecting degree of deviation of the outcome from the expectation) without separating positive or negative prediction error trials. Model prediction error values were then regressed against the fMRI data for each individual subject, to identify brain regions correlating with the model-predicted time series [22].

Condition Analysis. We developed first-level models to predict the response in each voxel as a function of each of five stimulus conditions: expected sucrose, unexpected sucrose, expected no-solution, unexpected no-solution, and expected artificial saliva. Two contrasts of interest were computed per subject: (1) unexpected sucrose receipt: trials with CS for no-solution followed by unexpected US sucrose contrasted against trials with CS for no-solution, followed by expected no-solution; (2) unexpected sucrose omission: trials with CS for sucrose solution followed by unexpected US no-solution contrasted against trials with CS for sucrose solution, followed by expected sucrose solution.

**Supplemental Table 1.**

|  |  | | | | | |  | | |  |  | | |
| --- | --- | --- | --- | --- | --- | --- | --- | --- | --- | --- | --- | --- | --- |
|  | **HC (N=46)** | | **AN (N=25)** | | **BN (N=21)** | | ***H*** | ***p*** | | **η^2^** |  | | |
|  | **Mean** | **SD** | **Mean** | **SD** | **Mean** | **SD** |  |  |  | | |  | |
| **Age** | 26.61 | 3.51 | 22.80 | 6.37 | 23.53 | 4.16 | 11.48 | 0.003 | 0.09 | | | HC>AN** | |
| **BMI** | 21.46 | 1.67 | 16.33 | 1.24 | 23.63 | 10.08 | 52.87 | <.001 | 0.55 | | | HC>AN*** | |
| **Body Dissatisfaction (EDI-3)** | 4.20 | 5.71 | 25.96 | 9.73 | 28.43 | 9.97 | 54.65 | <.001 | 0.57 | | | AN, BN>HC*** | |
| **Drive for Thinness (EDI-3)** | 0.90 | 1.11 | 5.56 | 7.61 | 17.48 | 7.70 | 45.14 | <.001 | 0.47 | | | BN>HC, AN*** | |
| **Bulimia (EDI-3)** | 1.83 | 2.92 | 18.52 | 9.46 | 20.81 | 5.62 | 52.84 | <.001 | 0.55 | | | AN, BN>HC*** | |
| **Low Self Esteem (EDI-3)** | 1.07 | 2.36 | 12.90 | 6.70 | 14.14 | 6.40 | 58.50 | <.001 | 0.62 | | | AN, BN>HC*** | |
| **Personal Alienation (EDI-3)** | 1.05 | 2.02 | 13.88 | 8.02 | 13.33 | 5.76 | 56.90 | <.001 | 0.60 | | | AN, BN>HC*** | |
| **Interpersonal Insecurity (EDI-3)** | 2.90 | 3.58 | 10.68 | 6.23 | 11.14 | 6.76 | 38.22 | <.001 | 0.39 | | | AN, BN>HC*** | |
| **Interpersonal Alienation (EDI-3)** | 2.12 | 3.68 | 10.04 | 5.76 | 10.81 | 5.87 | 39.40 | <.001 | 0.40 | | | AN, BN>HC*** | |
| **Interoceptive Deficits (EDI-3)** | 1.41 | 2.11 | 17.13 | 8.70 | 19.19 | 8.20 | 57.70 | <.001 | 0.61 | | | AN, BN>HC*** | |
| **Emotional Dysregulation (EDI-3)** | 0.54 | 1.19 | 7.64 | 6.47 | 8.57 | 6.42 | 45.22 | <.001 | 0.47 | | | AN, BN>HC*** | |
| **Perfectionism (EDI-3)** | 9.00 | 4.54 | 15.44 | 5.42 | 12.62 | 5.38 | 21.91 | <.001 | 0.21 | | | AN, BN>HC*** | |
| **Asceticism (EDI-3)** | 2.54 | 2.46 | 14.08 | 5.48 | 14.00 | 4.46 | 60.93 | <.001 | 0.64 | | | AN, BN>HC*** | |
| **Maturity Fears (EDI-3)** | 3.73 | 3.18 | 11.30 | 7.23 | 9.38 | 8.17 | 19.20 | <.001 | 0.18 | | | AN, BN>HC*** | |
| **State Anxiety (STAI)** | 25.48 | 7.19 | 57.52 | 11.89 | 58.05 | 14.01 | 55.28 | <.001 | 0.58 | | | AN, BN>HC*** | |
| **Trait Anxiety (STAI)** | 26.85 | 6.45 | 57.80 | 12.80 | 60.71 | 13.63 | 54.19 | <.001 | 0.57 | | | AN, BN>HC*** | |
| **Depression (BDI)** | 1.61 | 2.26 | 31.96 | 13.63 | 30.29 | 11.63 | 62.88 | <.001 | 0.67 | | | AN, BN>HC*** | |
| **PBI Care Mother** | 29.78 | 8.47 | 24.36 | 9.03 | 19.15 | 9.99 | 17.68 | <.001 | 0.19 | | | HC>AN*, BN*** | |
| **PBI Overprotection Mother** | 11.24 | 7.85 | 15.82 | 9.22 | 17.16 | 7.60 | 9.35 | 0.003 | 0.08 | | | BN>HC* | |
| **PBI Care Father** | 27.95 | 8.22 | 20.54 | 11.52 | 19.79 | 9.76 | 9.81 | 0.007 | 0.09 | | | HC>AN*, BN* | |
| **PBI Overprotection Father** | 9.15 | 8.21 | 12.91 | 6.82 | 14.39 | 8.10 | 11.55 | 0.009 | 0.11 | | | AN, BN>HC* | |
|  |  |  |  |  |  |  |  |  |  | | | |  |

Of the HC group, 17% were Asian, 2.4% Black or African American, 75.6% White, 2.4% White/Asian, and 2.4% Unknown race. Of the ED group, 2.2% were Asian, 8.7% Black or African American, 84.8% White, 2.2% White/American Indian, and 2.2% of Unknown race. Chi-Square test indicated no significant group difference (Pearson Chi-Square=10.96, p=0.09). Of the individuals in the HC group, 9.8%, and of the ED group 6.5% were of Latinx ethnicity, which was not significantly different (Pearson Chi-Square=0.307, p=0.580). In the AN group, 16 individuals (64%) were of the restricting and 9 (36%) of the binge eating/purging subtype.

**Supplemental Figure 1. Parental bonding across study groups.**

*** *p*<0.05, ****p*<0.001**

**Supplemental Figure 2. Mediation model.**
